# Concordance between a new molecular real-time approach and traditional culture in suspected VAP patients

**DOI:** 10.1186/cc14187

**Published:** 2015-03-16

**Authors:** M Clavel, O Barraud, V Moucadel, MC Ploy, E Karam, F Meynier, Study Group François for Valibi B

**Affiliations:** 1 Hopital Dupuytren, Limoges, France; 2 UMRS-1092, Hopital Dupuytren, Limoges, France; 3 Biomérieux SA, Grenoble, France; 4 Service de Réanimation, Brive, France; 5 Inserm, Limoges, France

## Introduction

Early microbiological documentation may reduce attributable mortality and excessive use of broad-spectrum antibiotics in ventilator-associated pneumonia (VAP). Using bronchoalveolar lavage (BAL) and endotracheal aspirates (ETA), we studied a new molecular biology-based approach to detect and quantify bacteria in less than 3 hours. This prospective multicenter trial aimed at comparing the microbiological results obtained using this molecular protocol (easyMAG® system) and semiquantitative culture in suspected VAP.

## Methods

ETA and BAL samples were consecutively collected during 10 months in adult patients in four ICUs of France. The molecular method includes a preprocessing liquefaction for ETA before DNA extraction. DNAs were extracted using the easyMAG® system. Real-time PCR (qPCR) was run using the ABI7500FastDx PCR instrument. The results presented here concern: *Staphylococcus aureus*, *Pseudomonas aeruginosa *and Enterobacteriaceae. Quantification was performed using qPCR standard curves, by converting the cycle threshold to CFU/ ml.

## Results

A total of 125 suspected VAP were included from 122 patients. In total, 125 BAL and 107 ETA were collected. Sex ratio (M/F) was 76%, and CPIS ≥6 was calculated in 74.6% of the suspected VAP patients. Mean ventilation duration before sampling was 6 days. Seventy-eight percent and 65% of the BAL and ETA culture were positive respectively. Correlations between molecular method and culture on BAL and ETA are reported in Table 1.

**Table 1:** Concordance between qPCR and culture on BAL/ETA in VAP patients.

Positive culture		qPCR	Agreement (%)	Sensitivity (%)	Specificity (%)
*S. aureus*(BAL/ETA)	28/20	31/25	96.7/89.7	96.6/76.9	96.8/93.8
*P. aeruginosa*(BAL/ETA)	23/20	20/23	97.6/93.5	100/100	97.1/92.4
Enterobacteriaceae (BAL/ETA)	27/7	36/18	90.3/85.0	90.0/58.3	90.4/88.4

## Conclusion

Sensitivity and specificity of the new molecular approach for these main bacteria found in VAP could enable targeted first-line antibiotic therapy. In the future, the development of this approach will aim at obtaining a bedside diagnostic in only a few hours.

